# The Association Between the Triglyceride Glucose Index and Hyperuricemia: A Dose–Response Meta-Analysis

**DOI:** 10.3390/nu17091462

**Published:** 2025-04-26

**Authors:** Juan Wang, Qiang He, Wenhui Sun, Wei Li, Yuting Yang, Weiwei Cui, Xiangshan Yang

**Affiliations:** 1 Department of Nutrition and Food Hygiene, School of Public Health, Jilin University, Changchun 130021, China; wangjuan23@mails.jlu.edu.cn (J.W.); sunwh23@mails.jlu.edu.cn (W.S.); liw23@mails.jlu.edu.cn (W.L.); ytyang23@mails.jlu.edu.cn (Y.Y.); cuiweiwei@jlu.edu.cn (W.C.); 2 Department of Radiation Hygiene, School of Public Health, Jilin University, Changchun 130021, China; heq@jlu.edu.cn

**Keywords:** triglyceride glucose index, hyperuricemia, dose–response meta-analysis

## Abstract

**Background:** The triglyceride glucose (TyG) index has been correlated with all kinds of diseases. However, its association with hyperuricemia is still a subject of controversy. **Methods:** This meta-analysis encompassed relevant studies on the TyG index and hyperuricemia obtained from electronic databases, from the launch date until March 2025. The effect sizes and corresponding 95% confidence intervals (CIs) were obtained using a random effects model. **Results:** Twenty-six trials with 637,954 subjects were incorporated in this study. It was revealed that the TyG index was linked to hyperuricemia (OR = 2.67; 95% CI: 2.34, 3.04; *p* < 0.001). A dose–response analysis demonstrated that with each 1 mg/dL rise in the TyG index, the risk of being diagnosed with hyperuricemia increased by 2.07 times (OR = 2.07; 95% CI: 1.89, 2.25; *p* < 0.001). **Conclusions:** The TyG index has an association with hyperuricemia. Given the constraints identified in our meta-analysis, further cohort studies will be essential to confirm this correlation.

## 1. Introduction

As a metabolic disorder, hyperuricemia stems predominantly from impaired purine metabolic pathways [1]. The core pathophysiological process involves excessive production or decreased excretion of uric acid (UA), resulting in elevated blood UA levels [2]. At the same time, the increased production or impaired excretion of UA leads to UA dysregulation, which in turn can lead to a variety of complications, and relevant research has shown that it is a risk factor for metabolic syndrome, diabetes and other related conditions [3,4,5]. From an epidemiological point of view, the incidence of hyperuricemia has tended to increase with rising standards of living and changing dietary habits [6]. The prevalence of hyperuricemia varies widely around the world, with reported rates ranging from 2.6% to 36%. Studies suggest that about 20% of adults in the United States have hyperuricemia, while the prevalence in China is about 14% [7,8]. This high prevalence of hyperuricemia is not only a threat to individual health, but also a growing public health problem.

The triglyceride glucose (TyG) index is an important biomarker of insulin resistance (IR), calculated as ln (triglycerides (TG) (mg/dL) × fasting plasma glucose (FPG) (mg/dL)/2) [9]. Meanwhile, regarding the pathogenic mechanism of hyperuricemia, several articles have shown that it is closely connected to IR; IR may contribute to the occurrence of hyperuricemia through several pathways, including increasing serum uric acid (SUA) levels, decreasing renal excretion of UA and causing renal damage [10,11,12]. IR is a metabolic disorder that occurs when tissues have a diminished response to insulin stimulation. This condition primarily affects glucose and lipid metabolism, leading to impairments in these processes [13]. Research has confirmed that IR has a strong association with various cerebrovascular diseases and their triggers, including atherosclerosis, the formation and rupture of carotid artery plaques, hyperglycemia, dyslipidemia and stroke [13,14,15,16]. It is widely recognized that both the hyperinsulinemic–euglycemic clamp technique and the Homeostatic Model Assessment of Insulin Resistance (HOMA-IR) are effective methods for measuring IR [17,18]. However, the hyperinsulinemic–euglycemic clamp technique is not ideal for routine clinical monitoring because it requires specialized equipment and personnel and is costly and complex to perform. HOMA-IR is calculated from fasting glucose and fasting insulin levels [19]. As insulin secretion capacity decreases, fasting insulin levels decrease and the calculated value of HOMA-IR decreases as a result of the lower insulin levels, which may underestimate IR and make it appear as if IR is decreasing. According to a previous study, the TyG index is positively correlated with IR. As the level of IR increases, the TyG index increases accordingly, and so it is proposed as a substitute indicator for evaluating IR. It has the advantages of being simple, cost-effective and credible [17,20]. Compared to the HOMA-IR, the TyG index can also be used to assess IR; its calculation does not depend on fasting insulin levels and is therefore less affected by the decline in insulin secretion capacity [21]. In addition, HOMA-IR requires the measurement of fasting insulin levels, which is a relatively expensive test, and the TyG index is more suitable for widespread use. Current evidence confirms that the TyG index has robust diagnostic efficacy for IR, with a sensitivity of 96.5% and specificity of 85.0% [22]. In particular, the TyG index has been shown to be better than HOMA-IR at predicting some conditions [23]. Additionally, the TyG index is not only strongly associated with IR, but is also associated with a higher risk of developing several cardiovascular diseases [9]. For example, the TyG index is related to the risk of heart failure, heart attack and atrial fibrillation [24,25]. These findings suggest that the TyG index is an important tool for identifying people at high risk of having cardiovascular disease [26,27].

IR has been linked to the pathogenic mechanisms of hyperuricemia. The TyG index is an inexpensive and credible index for assessing IR. Recently, more and more trials have presented results that TyG index is correlated with various metabolic diseases [28,29], including hyperuricemia, and that people with a high TyG index tend to have hyperuricemia. However, several studies have failed to find this association [30,31,32]. It remains uncertain what effect the TyG index has on hyperuricemia, and this association has not yet been shown in a meta-analysis. Thus, this study performed a meta-analysis by systematically collecting and analyzing all relevant studies to clarify the potential correlation between the TyG index and hyperuricemia and to build a more reliable basis for clinical practice and future research.

## 2. Methods

### 2.1. Data Sources and Retrieval Methods

This meta-analysis was designed following the relevant PRISMA guidelines (PROSPERO ID: CRD42023485721) [33], and its concrete contents are shown in the Table S1. We searched the literature linked to the TyG index and hyperuricemia in electronic databases such as PubMed, Google Scholar, Cochrane Library and the Web of Science from the launch date until March 2025. The literature search was conducted using the following keywords (combined with ‘OR’ or ‘AND’): triglyceride-glucose index, TyG index, TyGs, gout, hyperuricemia, HUA, HU and urate. Details of the search strategy are described in Table S2.

### 2.2. Criteria for Inclusion and Exclusion

**Figure 1:**
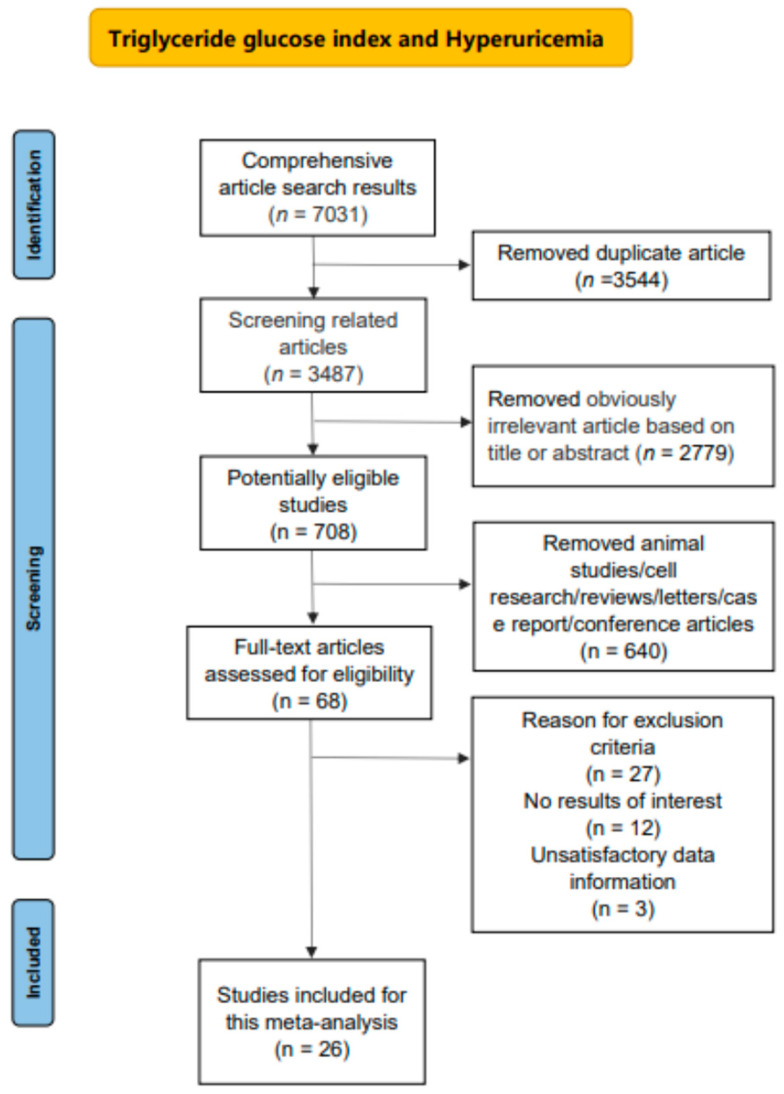
Flowchart for searching and selecting the literature.

These articles were selected using the subsequent criteria: (1) the TyG index was already calculated; (2) diagnosis of hyperuricemia was explicit; (3) the effect of the TyG index on hyperuricemia was described using the odds ratio (OR) or hazard ratio (HR) with 95% confidence intervals (CIs); (4) the article was published in English; (5) the included studies were conducted with human subjects; and (6) some of the literature, including duplicate studies, reviews and conference papers, was excluded. Two investigators assessed the relevant articles independently. They extracted qualified data and solved discrepancies with experts by the Delphi technique (Figure 1).

### 2.3. Data Extraction

Relevant data were systematically collected from the articles, including the first author, publication year, country, design, participant features, sample size, mean age, gender, diagnosis of hyperuricemia, adjusted confounding factors, TyG index, adjusted total effect estimates, effect sizes of subgroups (region, TyG index analysis, gender, age, body mass index, diabetes, estimate glomerular filtration rate (eGFR), hypertension, heart disease, year of publication and diagnosis of hyperuricemia) and their 95% CIs.

### 2.4. Quality Assessment

Two researchers evaluated the risk of bias in the articles via the Newcastle–Ottawa Scale (NOS) [34]. If the quality score was above 6, it was considered to have a low risk of bias [35]. The NOS comprises three components: selection, comparability and exposure. The selection and exposure sections of a study can only be awarded one star at most. The comparability section can be awarded a maximum of two stars. Additionally, the Grading of Recommendations Assessment, Development and Evaluation (GRADE) system was employed to appraise the quality of the evidence in the articles [36]. The articles were rated with four quality levels: high, moderate, low and very low.

### 2.5. Statistical Analysis

Statistical analyses were conducted using Rev Man 5.4 by Cochrane Collaboration (London, UK), SPSS 27.0 by IBM and Stata 12.0 by Stata Corp (College Station, TX, USA). Estimates and 95% CIs were obtained using the multivariate adjusted OR and HR for the included articles. The random effects model was utilized for this purpose. The assessment of statistical heterogeneity was conducted using Cochran’s Q statistic and the *I*^2^ statistic. If *p* < 0.05, significant heterogeneity was deemed present. When the *I*^2^ value was 25%, 50% and 75%, respectively, the degree of heterogeneity was low, medium and high [37]. We conducted a sensitivity analysis, meta-regression analyses and subgroup analyses to find the origins of heterogeneity. The meta-regression and subgroup analyses were designed on the basis of region (Asia and North America), TyG index analysis (continuous and categorized), gender (male and female), age (<60 and ≥60 years), body mass index (non-overweight and overweight), diabetes (yes and no), eGFR (<60 and ≥60 mL/min per 1.73 m^2^), hypertension (yes and no), heart diseases (yes and no), year of publication (<2023 and ≥2023) and diagnosis of hyperuricemia (7 mg/dL for men, 6 mg/dL for women, or other).

Funnel plots and Egger’s test were utilized for the assessment of the potential publication bias [38]. In addition, we conducted a dose–response analysis to study the connection between the TyG index and hyperuricemia [39]. In this analysis, the average or median given in the article was used as an estimate for this interval. For some studies that only provided ranges, we took the average of the high and low ends of the range as our estimate. Furthermore, in the case of the open interval, these studies divided the TyG index into four equal parts. The estimate for the first or fourth interval could be obtained by subtracting or adding the difference between the middle two estimates from the second or third interval estimate. It must be noted that all of these estimates fell within their respective interval.

## 3. Results

In total, 7031 relevant electronic database articles were screened using the search strategy. After our assessment, twenty-six articles (637,954 participants) including two cohort trials and twenty-five case-control/cross-sectional trials (with one article containing both cohort and cross-sectional parts) met the criteria, which are presented in Table 1. We evaluated the risk of bias via NOS, and the results are presented in Table 1, Tables S3 and S4. The average NOS score was 7.27 (low bias risk). Meanwhile, the quality of evidence in the eighteen trials was rated via the GRADE, and it showed that the quality of evidence was rated high for the case–control and cross-sectional studies (Table S5), and moderate for the cohort studies (Table S6).

**Figure 2:**
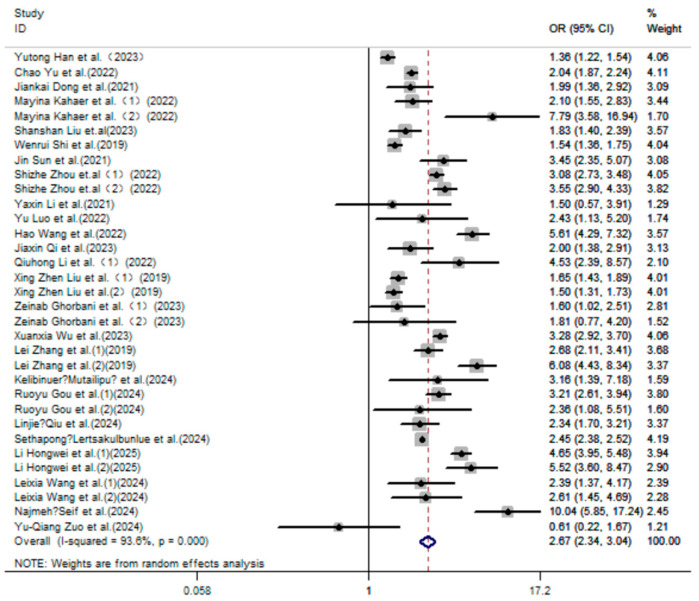
Forest plot of the risk of hyperuricemia in the high TyG index group versus the control group [2,30,31,32,40,41,42,43,44,45,46,47,48,49,50,51,52,53,55,56,57,58,59,60,61].

Our study included twenty-five case–control/cross-sectional studies with 591,933 participants to investigate the connection between the TyG index and hyperuricemia. The meta-analysis found that groups with higher values on the TyG index were more likely to have hyperuricemia than the groups with lower values on the TyG index (OR = 2.67; 95% CI: 2.34, 3.04; *p* < 0.001; Figure 2).

**Figure 3:**
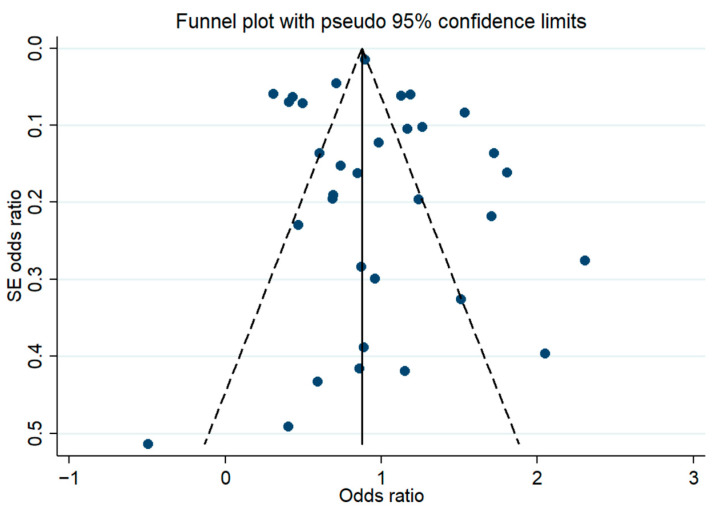
Funnel plot of TyG index effect estimates.

Moreover, we did not detect publication bias in the use of Egger’s test and the funnel plot (coefficient = 0.60, t = 0.67, *p* = 0.507, Figure 3). This ensured the accuracy and reliability of the findings and avoided erroneous conclusions due to selective publication.

The sources of heterogeneity were evaluated by a sensitivity analysis, meta-regression and subgroup analyses. The sensitivity analysis did not yield any significant findings (Figure S1). Meta-regression and subgroup analyses were performed based on region, TyG index analysis, gender, age, body mass index, diabetes, eGFR, hypertension, heart diseases, year of publication and diagnosis of hyperuricemia. In the subgroup analyses, the results indicated that the TyG index and hyperuricemia had a positive association (Table 2). Moreover, in the subgroup analyses based on the TyG index analysis (continuous and categorized), although both results declared that hyperuricemia was linked to the TyG index, we found that this correlation was more strong in the categorized TyG index (*p* < 0.001). Similarly, on the basis of gender, this study found a higher correlation for women than for men (*p* < 0.05). In the meta-regression analyses, we only found the TyG index classification to have an effect on the total effect (*p* < 0.05, Table 2).

**Figure 4:**
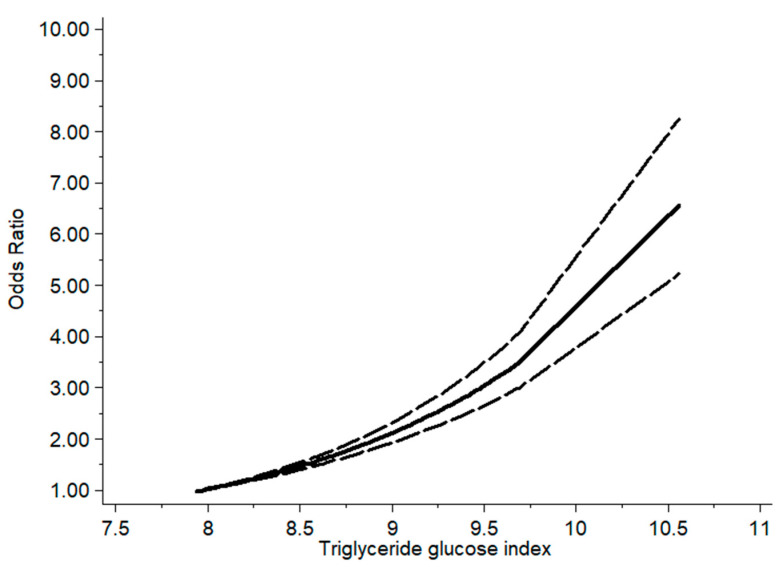
Dose–response plot of the TyG index and hyperuricemia (The solid line shows the dose-response relationship estimated by statistical modelling, and the dashed line shows the 95% CI for the solid line).

The dose–response analysis indicated the existence of a linear relationship between the TyG index and hyperuricemia (*p* > 0.05), and with each 1 mg/dL rise in TyG index, the risk of being diagnosed with hyperuricemia increased 2.07 times (OR = 2.07; 95% CI: 1.89, 2.25; *p* < 0.001) (Figure 4).

Two cohort studies with 46,021 subjects were included in our study. Groups with higher values on the TyG index had an increased risk of being diagnosed with hyperuricemia than groups with lower values on the TyG index (HR = 1.68; 95% CI: 1.30, 2.17; *p* < 0.05).

## 4. Discussion

Hyperuricemia is a prevalent disease on the global scale, especially in high- and middle-income countries. However, the incidence of the condition varies significantly across different geographical locations, with ethnicity, dietary habits and economic conditions all contributing to this variability [62,63]. Hyperuricemia has become a global health problem with changing lifestyles and the obesity epidemic [64]. The hyperuricemia incidence was 30.6 per 1000 person-years in a Chinese population-based study [54]. The hyperuricemia incidence was 31.7 per 1000 person-years in another study based on the Japanese population [65]. The significant correlation between hyperuricemia and IR, as measured by the TyG index, has been established through numerous experimental and epidemiological studies [66,67,68,69]. However, the direct connection between the TyG index and hyperuricemia is not certain. This meta-analysis displayed that the TyG index was linked to hyperuricemia, and with each 1 mg/dL rise in the TyG index, the risk of being diagnosed with hyperuricemia increased 2.07 times.

It is not yet clear what mechanism is involved in linking the TyG index to hyperuricemia. However, some mechanisms have shown that hyperuricemia is closely linked to IR. IR causes hyperuricemia through several pathways, including by elevating SUA levels, decreasing renal UA excretion and causing kidney damage [70]. ATP-binding cassette subfamily G member 2 (ABCG2) is mainly involved in UA secretion; urate transporter 1 (URAT1) is the major protein involved in UA reabsorption [71]. The regulation of UA excretion involves multiple transporters responsible for both secretion (ABCG2) and reabsorption (URAT1) [72,73]. During acute hyperinsulinemia resulting from IR, the body may alter the expression of these transporters to enhance urate reabsorption, thereby elevating SUA levels [72,73]. Research has indicated that glycolysis intermediates undergo conversion into 5-phosphoribose and phosphoric acid ribose pyrophosphate under IR. This process ultimately leads to increased SUA production [74]. Furthermore, compensatory hyperinsulinemia occurring after IR results in decreased UA excretion through the renal tubular reabsorption of sodium. Simultaneously, hyperinsulinemia activates the renin-angiotensin system, resulting in decreased renal blood flow, heightened urate reabsorption and stimulation of the production of xanthine oxidase. This results in an increase in the production of UA [5]. Conversely, hyperuricemia can cause IR and inflammation through effects on adipocytes and a reduction in mitochondrial oxidative stress and nitric oxide bioavailability [75]. A bidirectional mendelian randomization study [76] provided strong evidence that there was no association between fasting insulin concentrations and genetically determined serum urate concentrations, either by polygenic scoring or strong individual locus inference. In contrast, there was a positive association between serum urate concentrations and genetically determined fasting insulin concentrations. The polygenic score for fasting insulin was also found to correlate with the serum urate concentration in the UK Biobank. This confirms a unidirectional causal relationship between IR and hyperuricemia. Thus, these studies showed the effect of the TyG index on hyperuricemia, which is consistent with what we have found in our study.

The TyG index is calculated by measuring FPG and TG. An additional possible mechanism is via the blood glucose and lipid levels [51]. Hyperglycemia and hyperlipidemia have been reported to reduce glyceraldehyde-3-phosphate dehydrogenase activity, thereby increasing the synthesis of UA [77]. Moreover, TG can cause the narrowing or blockage of the small renal arteries due to long-term dyslipidemia, ultimately causing disorders in urate excretion. Research has revealed that TG levels are a risk factor for hyperuricemia [78]. Based on the information provided, and considering the significant diagnostic power of the TyG index for IR, we theorized an association between the TyG index and hyperuricemia.

This study showed that there was a connection between the TyG index and hyperuricemia in both men and women, but the connection seemed to be stronger in women, which may have been caused by differences in sex hormones and cytokines. Most of the subjects in this study were middle-aged and elderly. Premenopausal women have higher levels of estrogen, which increases their insulin sensitivity [79]. However, after menopause, estrogen levels fall and women have a significantly increased risk of IR [80]. Men have higher levels of androgens, which physiologically help to maintain insulin sensitivity [81]. In addition, almost all women go through menopause, but men do not [82]. Studies have shown that increased levels of cytokines, including TNF-α and IL-6, are associated with menopause [83], and these cytokines can contribute to IR by interfering with the insulin signaling pathway and interfering with the normal action of insulin [84]. Increases in IR can lead to a decrease in the kidneys’ ability to excrete UA, thereby increasing blood UA levels, which may be why we see this difference in gender.

This study had several advantages. Firstly, from what we know, it is the first meta-analysis study to discover the direct connection between the TyG index and hyperuricemia. Secondly, a sensitivity analysis and subgroup and meta-regression analyses were utilized to assess sources of heterogeneity. Thirdly, the mean NOS score of this study was 7.27 (low risk of bias). Concurrently, the results of the GRADE indicated that the quality of evidence derived from case–control and cross-sectional studies was classified as high. Finally, the linear relationship between the TyG index and hyperuricemia was investigated. This was performed using dose–response analysis. We found that the risk of being diagnosed with hyperuricemia increased by a factor of 2.07 for every 1 mg/dL increase in the TyG index, a finding that underlines the important role of the TyG index in assessing hyperuricemia. By monitoring the TyG index, high-risk groups can be identified at an early stage and targeted interventions can be implemented.

However, the limitations of our analysis must be considered. Firstly, the study was designed as a meta-analysis of observational trials due to the paucity of published articles and the temporary absence of relevant randomized controlled trials, so we could not conclude a causal connection between the TyG index and hyperuricemia, and the level of evidence was lower. Secondly, a considerable degree of heterogeneity was found among the studies, which may have been attributed to the existence of confounding factors. Due to the lack of information, we could not exclude the possibility that unadjusted residual factors may have confounded the connection between the TyG index and hyperuricemia, such as participants’ ethnicities, clinical comorbidities, alcohol consumption, the concurrent medications used and so on. Therefore, a larger number of trials are needed for evaluation. Finally, there were only two cohort studies, and the studies differed widely in how many participants they sampled.

## 5. Conclusions

Based on the meta-analysis conducted, the TyG index has an association with hyperuricemia. Meanwhile, this analysis revealed that the risk of being diagnosed with hyperuricemia increased by a factor of 2.07 for every 1 mg/dL increase in the TyG index. The TyG index can be used as a complementary indicator for the comprehensive assessment of health and disease risk, providing more valuable information for clinical decision-making. Nevertheless, the limitations of our analysis must be considered. Cohort studies will be necessary for further investigating the causal connection between the TyG index and hyperuricemia. More subgroup analyses are recommended for any type of study. In addition, the combined effect of the TyG index with other metabolic syndrome-related indices on hyperuricemia and their predictive value in different populations could be further investigated.

## Figures and Tables

**Table 1:** Characteristics of included trials.

Study	Country	Design	Participant Features	Sample Size	Mean Age (Years)	Male (%)	TyG Index Analysis	Diagnosis of Hyperuricemia	Variables Adjusted	NOS
Yutong Han et al. [40]	China	case–control study	participants aged ≥ 45 years	5269	58.58 ± 8.61	45.28	Continuous; Categorized (Q4:Q1)	SUA ≥ 7 mg/dL (men) and ≥6 mg/dL (women)	age, gender, residence, education, marital status, smoking, drinking, HTN, diabetes, CVD, dyslipidemia and TC, BUN, Cre, HbA1c and C-reactive proteins	8
Chao Yu et al. [41]	China	cross-sectional study	adults with HTN	13,060	63.81	51.04	Continuous; Categorized (Q4:Q1)	SUA ≥ 7 mg/dL	gender, age, BMI, SBP, DBP, education, exercise, WC, drinking, smoking, HDL-C, LDL-C, serum homocysteine, eGFR, diabetes and antiplatelet and antihypertensive medicines	6
Jiankai Dong et al. [42]	China	cross-sectional study	in-patients with primary hypertension	428	67.86 ± 6.96	46.70	Continuous	two non-daily fasting SUA levels ≥ 7 mg/dL (men) and ≥6 mg/dL (women)	gender, age, body weight, smoking, drinking and BMI	6
Mayina Kahaer et al. [43]	China	cross-sectional study	the medical checkup population	2243	41.55 ± 12.70	72.05	Categorized (Q4:Q1)	SUA > 7 mg/dL	age, SBP, DBP, BUN, Cre, TC and LDL-C	7
Shanshan Liu et al. [44]	China	cross-sectional study	in-patients with primary HTN	1707	62.97 ± 12.87	46.00	Continuous; Categorized (Q3:Q1)	SUA ≥ 7 mg/dL	age, gender, ALB, ALT, AST, Scr, BUN, d-dimer, INR, eGFR, HTN, LDL-C, HDL-C and LPa	6
Wenrui Shi et al. [45]	China	cross-sectional study	general population	6466	59.57 ± 10.49	39.81	Continuous; Categorized (Q4:Q1)	SUA ≥ 7 mg/dL (men) and ≥6 mg/dL (women)	age, gender, education, income, exercise, smoking, drinking, BMI, HTN, DM, eGFR, HDL-C, LDL-C, antidiabetic and lipid-lowering therapy and CVD	8
Jin Sun et al. [46]	China	cross-sectional study	community-based	4551	58.63 ± 8.33	33.60	Categorized (Q4:Q1)	SUA ≥ 7 mg/dL (men) and ≥6 mg/dL (women)	age, gender, SBP, DBP, Scr, BUN, stroking, CHD and DM, serum cholesterol, HDL-C, LDL-C, BMI, WC and hip circumference	8
Shizhe Zhou et al. [47]	China	cross-sectional study	college students	23,411	18.28 ± 0.64	47.74	Categorized (Q4:Q1)	two measurements on different days, SUA > 7 mg/dL	age, SBP, DBP, BUN, Cre, ALT, AST and TC	7
Yaxin Li et al. [30]	China	cross-sectional study	population-based community	4352	-	44.97	Categorized (Q4:Q1)	SUA ≥ 7 mg/dL (men) and ≥6 mg/dL (premenopausal women)	gender, age, education, smoking, drinking, exercise, TC, LDL-C and eGFR	8
Yu Luo et al. [48]	China	cross-sectional study	patients with T2DM	719	-	60.64	Continuous	SUA > 7 mg/dL (men) and >6 mg/dL (women)	age, gender, BMI, ALB, ALT, AST, BUN, Scr, TG, HDL-C, FPG, HbA1c and fatty liver	6
Hao Wang et al. [49]	US	cross-sectional study	non-diabetic patients	7743	45.17 ± 17.10	49.15	Categorized (Q4:Q1)	SUA ≥ 6 mg/dL	gender, age, race, education, smoking, drinking, SBP, DBP, MET, TC, LDL-C and eGFR	8
Jiaxin Qi et al. [50]	China	retrospective case–control study	patients with NAFLD	461	-	41.20	Continuous; Categorized (Q3:Q1)	SUA > 7 mg/dL (men) and >6 mg/dL (women)	age, gender, BMI, HTN, DM, smoking, ALT, AST and Scr	6
Qiuhong Li et al. (1) [51]	China	cross-sectional study	patients with diabetic nephropathy	6471	59.11 ± 10.53	58.41	Categorized (Q4:Q1)	SUA ≥ 7 mg/dL	age, gender, HDL-C, LDL-C, BMI, eGFR, 24hTP, SBP, DBP and HbA1c	6
Qiuhong Li et al. (2) [51]	China	cohort study	patients with diabetic nephropathy	3634	-	-	Categorized (Q4:Q1)	SUA ≥ 7 mg/dL	age, gender, HDL-C, LDL-C, BMI, eGFR, 24hTP, SBP, DBP and HbA1c	8
Xing Zhen Liu et al. [52]	China	cross-sectional study	adults without self-reported use of drugs	174,695	45.00 ± 12.20	60.20	Categorized (Q4:Q1)	SUA ≥ 7 mg/dL (men and postmenopausal women) and ≥6 mg/dL (premenopausal women)	age, smoking, WC and eGFR	7
Zeinab Ghorbani et al. [32]	Iran	cross-sectional study	individuals who visited the cardiology outpatient clinic	1170	-	40.60	Categorized (Q3:Q1)	SUA ≥ 5.6 mg/dL	gender; age; HTN, T2DM or hyperlipidemia; using antihypertensive α, antidiabetic β or antihyperlipidemic medications γ; and smoking	6
Xuanxia Wu et al. [53]	China	cross-sectional study	general population	32,354	-	55.94	Categorized (Q4:Q1)	SUA > 7 mg/dL (men) and >6 mg/dL (women)	gender, age, race, residence, marital status, BMI, abdominal obesity, HTN, diabetes, CHD and dyslipidemia	8
Qing Gu et al. [54]	China	cohort study	general population	42,387	43.10 ± 12.30	56.30	Categorized (Q3:Q1)	SUA ≥ 7 mg/dL (men and postmenopausal women) and ≥6 mg/dL (premenopausal women or those receiving urate lowering therapies)	age, smoking, BMI, HTN, NAFLD, eGFR and urate	9
Lei Zhang et al. [55]	China	cross-sectional study	participants of physical examination	24,438	47.23	51.38	Categorized (Q4:Q1)	SUA ≥ 7.392 mg/dL (men) and ≥6 mg/dL (women)	age, alanine aminotransferase, γ-glutamyl transpeptidase, Scr, BUN, TC and HDL-C	8
Kelibinuer Mutailipu et al. [56]	China	cross-sectional study	Department of Endocrinology at the hospital	951	31.00	42.69	Categorized (Q4:Q1)	SUA ≥ 7 mg/dL (men) and >6 mg/dL (women)	age, HR, HbA1c, FPG, TC, TG, HDL, LDL, BAI and LAP	6
Ruoyu Gou et al. [2]	US and China	cross-sectional study	data from NHANES in US and CHARLS in China	US: 14,259 China: 4613	US: 45.92 China: 68.52	US: 52.67 China: 69.91	Categorized (Q4:Q1)	SUA ≥ 7 mg/dL (men) and ≥6 mg/dL (women)	gender, age, marital status, education, HTN, diabetes, hypertriglyceridemia and healthy lifestyle score	8
Yu-Qiang Zuo et al. [31]	China	cross-sectional study	an annual health check-up population	6219	39.13	22.77	Categorized (Q4:Q1)	two non-fasting SUA levels ≥ 7 mg/dL	gender, age, drinking, smoking, menopause status, LDL-C and TC.	8
Linjie Qiu et al. [57]	US	cross-sectional study	data from the NHANES	8572	49.2	49.93	Continuous	SUA ≥ 7 mg/dL (men) and ≥6 mg/dL (women)	age, gender, race, education, marital status, smoking, drinking, exercise, BMI, family income to poverty ratio, LDL, HDL, HbA1c, Scr, eGFR, HTN, diabetes, arthritis, CHD and stroke	8
Sethapong Lertsakulbunlue et al. [58]	Thailand	cross-sectional study	Royal Thai Army personnel	231,286	47.4	89.4	Categorized (Q4:Q1)	SUA ≥ 7 mg/dL (men) and ≥6 mg/dL (women)	age, gender, BMI, region, scheme, year, smoking, drinking, exercise, SBP, DBP, AST and ALT	6
Li Hongwei et al. [59]	China	cross-sectional study	adults undergoing health screening	14,834	50.6	65.98	Categorized (Q4:Q1)	a fasting SUA > 7 mg/dL (men), and > 6 mg/dL (women)	age, SBP, DBP, FPG, smoking, drinking, exercise and diet.	8
Leixia Wang et al. [60]	US	cross-sectional study	data from the NHANES	7367	51.8	48.34	Categorized (Q4:Q1)	SUA ≥ 7 mg/dL (men) and ≥6 mg/dL (women)	gender, age, education, race, smoking, drinking, exercise, BMI, WC, TC, TG, HDL-C, LDL-C, HbA1c, fasting blood glucose and self-reported comorbidities	8
Najmeh Seif et al. [61]	Iran	cross-sectional study	part of the Mashhad Stroke and Heart Atherosclerotic Disorder cohort study	6457	48.44	39.94	Categorized (Q4:Q1)	SUA ≥ 7 mg/dL (men) and ≥6 mg/dL (women)	age, gender, BMI, energy intake, education, smoking, exercise, chronic diseases including diabetes, HTN, dyslipidemia and eGFR.	8

NOS, Newcastle–Ottawa scale; TyG, triglyceride glucose index; BMI, body mass index; SBP, systolic blood pressure; eGFR, estimate glomerular filtration rate; DBP, diastolic blood pressure; LDL-C, low-density lipoprotein cholesterol; TG, plasma triglyceride level; TC, total cholesterol; HDL-C, high-density lipoprotein cholesterol; AST, aspartate aminotransferase; ALT, alanine aminotransferase; SUA, serum uric acid; MET, metabolic equivalent value; BUN, blood urea nitrogen; Cre, creatinine; ALB, albumin; Scr, serum creatinine; INR, international normalized ratio; LPa, lipoprotein a; DM, diabetes mellitus; CVD, cardiovascular disease; FPG, fasting plasma glucose; HbA1c, glycosylated hemoglobin; HTN, hypertension; 24hTP, 24 h total urine protein; WC, waist circumference; T2DM, type 2 diabetes mellitus; CHD, coronary heart disease; NAFLD, nonalcoholic fatty liver disease; NHANES, National Health and Nutrition Examination Survey.

**Table 2:** Summary of the results of the subgroup and meta-regression analyses.

Subgrouped by	No. of Trials	OR	95% CI	*I*^2^ (%)	*P*_over effect_	*P*_interaction_	*P*_meta-regression_
region	25	2.67	(2.34, 3.04)	93.6	<0.001	0.43	0.495
Asia	22	2.58	(2.24, 2.98)	94.1	<0.001		
North America	4	3.15	(2.22, 4.47)	61.3	<0.001		
TyG index analysis	25	2.67	(2.34, 3.04)	93.6	<0.001	<0.001	0.017
continuous	8	1.82	(1.54, 2.14)	81.6	<0.001		
categorized	17	3.05	(2.61, 3.56)	92.6	<0.001		
gender	13	2.60	(2.22, 3.05)	92.6	<0.001	0.03	0.053
men	12	2.21	(1.80, 2.70)	92.9	<0.001		
women	13	3.18	(2.43, 4.16)	92.8	<0.001		
age	8	2.16	(1.78, 2.63)	87.7	<0.001	0.18	0.222
<60	4	2.41	(1.72, 3.38)	93.5	<0.001		
≥60	7	1.89	(1.69, 2.11)	13.3	0.328		
body mass index	7	1.82	(1.59, 2.08)	84.4	<0.001	0.18	0.241
non-overweight	3	1.62	(1.31, 2.00)	74.4	0.008		
overweight	6	1.94	(1.66, 2.26)	79.3	<0.001		
diabetes	8	2.20	(1.55, 3.13)	87.1	<0.001	0.81	0.837
yes	4	2.09	(1.10, 3.98)	81.7	0.001		
no	6	2.30	(1.52, 3.48)	87.9	<0.001		
eGFR	3	1.78	(1.50, 2.11)	74.6	0.003	0.60	0.637
<60	2	1.66	(1.28, 2.17)	0.0	0.540		
≥60	3	1.82	(1.46, 2.27)	86.1	0.001		
hypertension	9	1.99	(1.73, 2.29)	66.8	<0.001	0.87	0.893
yes	8	1.98	(1.74, 2.26)	52.2	0.033		
no	5	1.92	(1.27, 2.89)	80.2	<0.001		
heart disease	3	2.08	(1.77, 2.44)	0.0	0.618	0.15	0.246
yes	2	1.61	(1.10, 2.36)	0.0	0.955		
no	3	2.19	(1.84, 2.62)	0.0	0.749		
year of publication	25	2.67	(2.34, 3.04)	93.6	<0.001	0.79	0.770
<2023	12	2.73	(2.21, 3.37)	93.8	<0.001		
≥2023	13	2.62	(2.15, 3.21)	93.5	<0.001		
diagnosis of hyperuricemia	25	2.67	(2.34, 3.04)	93.6	<0.001	0.43	0.597
1 *	16	2.55	(2.13, 3.04)	94.7	<0.001		
other	9	2.87	(2.26, 3.64)	91.2	<0.001		

* SUA: 7 mg/dL for men and 6 mg/dL for women.

## Data Availability

The published article and its Supplementary Information files contain all data generated or analyzed during this trial.

## Supplementary Materials

The following supporting information can be downloaded at: https://www.mdpi.com/article/10.3390/nu17091462/s1, Figure S1: The sensitivity analysis of the included studies. Table S1: PRISMA 2020 checklist. Table S2: The detailed search strategies. Table S3: Risk of bias in case–control/cross-sectional studies by NOS. Table S4: Risk of bias in cohort studies by NOS. Table S5: Summary of the results using the GRADE (case–control/cross-sectional studies). Table S6: Summary of the results using the GRADE (cohort studies).
