# Supplementary material for: The Association Between the Triglyceride Glucose Index and Hyperuricemia: A Dose–Response Meta-Analysis
Source: Nutrients. 2025 Apr 26;17(9):1462. doi: 10.3390/nu17091462 (PMC12073563; doi:10.3390/nu17091462)
Supplement: Supplementary file 1 [file nutrients-17-01462-s001.zip › nutrients-3561353-supplementary.pdf]

Table S1: PRISMA 2020 Checklist

Section and Topic	Item #	Checklist item	Location where item is reported
**TITLE**			
Title	1	Identify the report as a systematic review.	1
**ABSTRACT**			
Abstract	2	See the PRISMA 2020 for Abstracts checklist.	1
**INTRODUCTION**			
Rationale	3	Describe the rationale for the review in the context of existing knowledge.	1-2
Objectives	4	Provide an explicit statement of the objective(s) or question(s) the review addresses.	2
**METHODS**			
Eligibility criteria	5	Specify the inclusion and exclusion criteria for the review and how studies were grouped for the syntheses.	2-3
Information sources	6	Specify all databases, registers, websites, organisations, reference lists and other sources searched or consulted to identify studies. Specify the date when each source was last searched or consulted.	2
Search strategy	7	Present the full search strategies for all databases, registers and websites, including any filters and limits used.	2
Selection process	8	Specify the methods used to decide whether a study met the inclusion criteria of the review, including how many reviewers screened each record and each report retrieved, whether they worked independently, and if applicable, details of automation tools used in the process.	2-3

Data collection process	9	Specify the methods used to collect data from reports, including how many reviewers collected data from each report, whether they worked independently, any processes for obtaining or confirming data from study investigators, and if applicable, details of automation tools used in the process.	3
Data items	10a	List and define all outcomes for which data were sought. Specify whether all results that were compatible with each outcome domain in each study were sought (e.g. for all measures, time points, analyses), and if not, the methods used to decide which results to collect.	3
	10b	List and define all other variables for which data were sought (e.g. participant and intervention characteristics, funding sources). Describe any assumptions made about any missing or unclear information.	3
Study risk of bias assessment	11	Specify the methods used to assess risk of bias in the included studies, including details of the tool(s) used, how many reviewers assessed each study and whether they worked independently, and if applicable, details of automation tools used in the process.	3-4
Effect measures	12	Specify for each outcome the effect measure(s) (e.g. risk ratio, mean difference) used in the synthesis or presentation of results.	4
Synthesis methods	13a	Describe the processes used to decide which studies were eligible for each synthesis (e.g. tabulating the study intervention characteristics and comparing against the planned groups for each synthesis (item #5)).	4
	13b	Describe any methods required to prepare the data for presentation or synthesis, such as handling of missing summary statistics, or data conversions.	4
	13c	Describe any methods used to tabulate or visually display results of individual studies and syntheses.	4
	13d	Describe any methods used to synthesize results and provide a rationale for the choice(s). If meta-analysis was performed, describe the model(s), method(s) to identify the presence and extent of statistical heterogeneity, and software package(s) used.	4
	13e	Describe any methods used to explore possible causes of heterogeneity among study results (e.g. subgroup analysis, meta-regression).	4
	13f	Describe any sensitivity analyses conducted to assess robustness of the synthesized results.	4
Reporting bias assessment	14	Describe any methods used to assess risk of bias due to missing results in a synthesis (arising from reporting biases).	3-4
Certainty assessment	15	Describe any methods used to assess certainty (or confidence) in the body of evidence for an outcome.	4

**RESULTS**			
Study selection	16a	Describe the results of the search and selection process, from the number of records identified in the search to the number of studies included in the review, ideally using a flow diagram.	Figure 1
	16b	Cite studies that might appear to meet the inclusion criteria, but which were excluded, and explain why they were excluded.	4
Study characteristics	17	Cite each included study and present its characteristics.	Table 1
Risk of bias in studies	18	Present assessments of risk of bias for each included study.	4
Results of individual studies	19	For all outcomes, present, for each study: (a) summary statistics for each group (where appropriate) and (b) an effect estimate and its precision (e.g. confidence/credible interval), ideally using structured tables or plots.	Table 1
Results of syntheses	20a	For each synthesis, briefly summarise the characteristics and risk of bias among contributing studies.	4, 12,14
	20b	Present results of all statistical syntheses conducted. If meta-analysis was done, present for each the summary estimate and its precision (e.g. confidence/credible interval) and measures of statistical heterogeneity. If comparing groups, describe the direction of the effect.	12-14
	20c	Present results of all investigations of possible causes of heterogeneity among study results.	13
	20d	Present results of all sensitivity analyses conducted to assess the robustness of the synthesized results.	13
Reporting biases	21	Present assessments of risk of bias due to missing results (arising from reporting biases) for each synthesis assessed.	12
Certainty of evidence	22	Present assessments of certainty (or confidence) in the body of evidence for each outcome assessed.	4
**DISCUSSION**			
Discussion	23a	Provide a general interpretation of the results in the context of other evidence.	14

	23b	Discuss any limitations of the evidence included in the review.	15-16
	23c	Discuss any limitations of the review processes used.	15-16
	23d	Discuss implications of the results for practice, policy, and future research.	14-15
**OTHER INFORMATION**			
Registration and protocol	24a	Provide registration information for the review, including register name and registration number, or state that the review was not registered.	2
	24b	Indicate where the review protocol can be accessed, or state that a protocol was not prepared.	N/A
	24c	Describe and explain any amendments to information provided at registration or in the protocol.	N/A
Support	25	Describe sources of financial or non-financial support for the review, and the role of the funders or sponsors in the review.	16
Competing interests	26	Declare any competing interests of review authors.	16
Availability of data, code and other materials	27	Report which of the following are publicly available and where they can be found: template data collection forms; data extracted from included studies; data used for all analyses; analytic code; any other materials used in the review.	Supplementary materials

Table S2 The detailed search strategies

#1	triglyceride-glucose index OR triglyceride glucose index OR TyG index OR triglyceride and glucose index OR triglyceride – glucose (T/Gly) index OR TyGs OR triglyceride glucose indices OR The triglyceride-glucose index OR Triglyceride/glucose index OR Triglycerides and glucose index
#2	gout or gouty or hyperuricemia or hyperuricaemia or uric acid or HUA or HU or urate or hyperuric or Trihydroxypurine or Trioxopurine or uricemia or serum uric acid or serum urate
#3	#1 AND #2

Table S3 The risk of bias for case-control/cross-sectional studies by NOS

Yutong Han et al. 2023

Study type	case-control study
Participants	participants aged ≥45 years, and they did not have hyperuricemia or kidney diseases at baseline.<br><br>Sample size:5269<br><br>Mean age in years: 58.58 ± 8.61<br><br>Gender: 2386males/2883females<br><br>Location: China
Outcomes	Main study outcome: The four IR surrogates have comparable predictive ability for hyperuricemia.<br><br>Available outcomes: Increased baseline values of TyG was significantly associated with higher risks of hyperuricemia.

Risk of bias

**Bias**	**Authors' judgment**	**Support for judgment**
Is the case definition adequate(Selection)	1	yes, with independent validation
Representativeness of the cases(Selection)	1	consecutive or obviously<br><br>representative series of cases
Selection of Controls(Selection)	1	community controls
Definition of Controls(Selection)	1	no hyperuricemia
Comparability of cases and controls on the basis of the design or analysis(Comparability)	2	study controls for age, gender and<br><br>other factors
Ascertainment of exposure(Exposure)	1	secure record (Laboratory<br><br>examination)

Same method of ascertainment for cases and controls(Exposure)	1	yes
Non-Response rate(Exposure)	0	non respondents described

Chao Yu et al. 2022

Study type	cross-sectional study
Participants	adults (aged 18 years or over) with hypertension<br>Sample size: 13060<br>Mean age in years:63.81<br>Gender: 6666males/6394females<br>Location:China
Outcomes	Main study outcome: Positive associations were found between the TyG index and serum uric acid and between the TyG index and hyperuricemia in adults with hypertension.<br>Available outcomes: the associations between the triglyceride glucose index and hyperuricemia.

Risk of bias

**Bias**	**Authors' judgment**	**Support for judgment**
Is the case definition adequate(Selection)	1	yes, with independent validation
Representativeness of the cases(Selection)	0	potential for selection biases(adults with hypertension)
Selection of Controls(Selection)	0	hospital controls
Definition of Controls(Selection)	1	no hyperuricemia
Comparability of cases and controls on the basis of the	2	study controls for age, gender and

design or analysis(Comparability)		other factors
Ascertainment of exposure(Exposure)	1	secure record (Laboratory examination)
Same method of ascertainment for cases and controls(Exposure)	1	yes
Non-Response rate(Exposure)	0	non respondents described

Jiankai Dong et al.2021

Study type	cross-sectional study
Participants	in-patients with primary hypertension<br>Sample size: 428<br>Mean age in years: 67.86 ± 6.96<br>Gender: 185males/243females<br>Location:China
Outcomes	Main study outcome: TYG index in elderly hypertensive patients is closely related to hyperuricemia, and the increase of TYG index is an independent risk factor for HUA in elderly hypertensive patients.<br>Available outcomes: the associations between the triglyceride glucose index and hyperuricemia.

Risk of bias

Bias	Authors' judgment	Support for judgment
Is the case definition adequate(Selection)	1	yes, with independent validation
Representativeness of the cases(Selection)	0	potential for selection biases(in-patients with primary hypertension)
Selection of Controls(Selection)	0	hospital controls

Definition of Controls(Selection)	1	no hyperuricemia
Comparability of cases and controls on the basis of the design or analysis(Comparability)	2	study controls for age, gender and<br><br>other factors
Ascertainment of exposure(Exposure)	1	secure record (Laboratory<br><br>examination)
Same method of ascertainment for cases and controls(Exposure)	1	yes
Non-Response rate(Exposure)	0	non respondents described

Mayina Kahaer et al. 2022

Study type	cross-sectional study
Participants	the medical checkup population<br><br>Sample size: 2243<br><br>Mean age in years:41.55 ± 12.70<br><br>Gender: 1616males/627females<br><br>Location:China
Outcomes	Main study outcome: The TyG index was significantly related to HUA and was superior to obesity indices in identifying HUA in the medical checkup population in Xinjiang, China.<br><br>Available outcomes: the associations between the triglyceride<br><br>glucose index and hyperuricemia.

Risk of bias

**Bias**	**Authors' judgment**	**Support for judgment**
Is the case definition adequate(Selection)	1	yes, with independent validation

Representativeness of the cases(Selection)	1	consecutive or obviously representative series of cases
Selection of Controls(Selection)	1	community controls
Definition of Controls(Selection)	1	no hyperuricemia
Comparability of cases and controls on the basis of the design or analysis(Comparability)	1	study controls for age and other factors
Ascertainment of exposure(Exposure)	1	secure record (Laboratory examination)
Same method of ascertainment for cases and controls(Exposure)	1	yes
Non-Response rate(Exposure)	0	non respondents described

Shanshan Liu et al. 2023

Study type	cross-sectional study
Participants	in-patients with primary hypertension<br>Sample size: 1707<br>Mean age in years:62.97 ± 12.87<br>Gender: 786males/921females<br>Location:China
Outcomes	Main study outcome: The TyG index was positively associated with HUA in patients with hypertension.<br>Available outcomes: the associations between the triglyceride glucose index and hyperuricemia.

Risk of bias

Bias	Authors' judgment	Support for judgment

Is the case definition adequate(Selection)	1	yes, with independent validation
Representativeness of the cases(Selection)	0	potential for selection biases(in-patients with primary hypertension)
Selection of Controls(Selection)	0	hospital controls
Definition of Controls(Selection)	1	no hyperuricemia
Comparability of cases and controls on the basis of the design or analysis(Comparability)	2	study controls for age, gender and<br><br>other factors
Ascertainment of exposure(Exposure)	1	secure record (Laboratory<br><br>examination)
Same method of ascertainment for cases and controls(Exposure)	1	yes
Non-Response rate(Exposure)	0	non respondents described

Wenrui Shi et al. 2019

Study type	cross-sectional study
Participants	a general Population<br><br>Sample size: 6466<br><br>Mean age in years:59.57 ± 10.49<br><br>Gender:2574males/3892females<br><br>Location:China
Outcomes	Main study outcome: the linear and robust association between TyG and hyperuricemia.<br><br>Available outcomes: the associations between the triglyceride<br><br>glucose index and hyperuricemia.

Risk of bias

Bias	Authors' judgment	Support for judgment
Is the case definition adequate(Selection)	1	yes, with independent validation
Representativeness of the cases(Selection)	1	consecutive or obviously representative series of cases
Selection of Controls(Selection)	1	community controls
Definition of Controls(Selection)	1	no hyperuricemia
Comparability of cases and controls on the basis of the design or analysis(Comparability)	2	study controls for age, gender and other factors
Ascertainment of exposure(Exposure)	1	secure record (Laboratory examination)
Same method of ascertainment for cases and controls(Exposure)	1	yes
Non-Response rate(Exposure)	0	non respondents described

Jin Sun et al. 2021

Study type	cross-sectional study
Participants	community-based<br>Sample size: 4551<br>Mean age in years:58.63±8.33<br>Gender:1531males/3020females<br>Location:China
Outcomes	Main study outcome: TyG index is significantly associated with hyperuricemia in hypertension patients among Han Chinese, obesity plays a partial mediation role in this relationship.

	Available outcomes: the associations between the triglyceride glucose index and hyperuricemia.

Risk of bias

Bias	Authors' judgment	Support for judgment
Is the case definition adequate(Selection)	1	yes, with independent validation
Representativeness of the cases(Selection)	1	consecutive or obviously representative series of cases
Selection of Controls(Selection)	1	community controls
Definition of Controls(Selection)	1	no hyperuricemia
Comparability of cases and controls on the basis of the design or analysis(Comparability)	2	study controls for age, gender and other factors
Ascertainment of exposure(Exposure)	1	secure record (Laboratory examination)
Same method of ascertainment for cases and controls(Exposure)	1	yes
Non-Response rate(Exposure)	0	non respondents described

Shizhe Zhou et al. 2022

Study type	cross-sectional study
Participants	college students<br>Sample size: 23411<br>Mean age in years:18.28±0.64<br>Gender: 11177males/12234females

	Location:China
Outcomes	Main study outcome: In clinical practice, LAP, TYG, and their related derivatives may be used as sensitive indicators for HUA prediction in college students.<br><br>Available outcomes: the associations between the triglyceride glucose index and hyperuricemia.

Risk of bias

Bias	Authors' judgment	Support for judgment
Is the case definition adequate(Selection)	1	yes, with independent validation
Representativeness of the cases(Selection)	1	consecutive or obviously representative series of cases
Selection of Controls(Selection)	1	community controls
Definition of Controls(Selection)	1	no hyperuricemia
Comparability of cases and controls on the basis of the design or analysis(Comparability)	1	study controls for age and other factors
Ascertainment of exposure(Exposure)	1	secure record (Laboratory examination)
Same method of ascertainment for cases and controls(Exposure)	1	yes
Non-Response rate(Exposure)	0	non respondents described

Yaxin Li et al. 2021

Study type	cross-sectional study
Participants	population-based community<br>Sample size:4352<br>Mean age in years:NA<br>Gender: 1957males/2395females<br>Location:China
Outcomes	Main study outcome: : The present study suggested that TyG index, TyG-BMI, TG/HDL-C and METS-IR had a significant correlation with hypertension plus hyperuricemia, and TyG-BMI and METS-IR had discriminative abilities for hypertension plus hyperuricemia.<br>Available outcomes: the associations between the triglyceride glucose index and hyperuricemia.

Risk of bias

Bias	Authors' judgment	Support for judgment
Is the case definition adequate(Selection)	1	yes, with independent validation
Representativeness of the cases(Selection)	1	consecutive or obviously representative series of cases
Selection of Controls(Selection)	1	community controls
Definition of Controls(Selection)	1	no hyperuricemia
Comparability of cases and controls on the basis of the design or analysis(Comparability)	2	study controls for age, gender and other factors
Ascertainment of exposure(Exposure)	1	secure record (Laboratory examination)

Same method of ascertainment for cases and controls(Exposure)	1	yes
Non-Response rate(Exposure)	0	non respondents described

Yu Luo et al. 2022

Study type	cross-sectional study
Participants	patients with T2DM<br><br>Sample size: 719<br><br>median age: 58<br><br>Gender: 436males/283females<br><br>Location:China
Outcomes	Main study outcome: TyG was positively correlated with SUA in non-obese T2DM patients. TyG may better predict HUA in non-obese T2DM patients than HOMA-IR.<br><br>Available outcomes: the associations between the triglyceride glucose index and hyperuricemia.

Risk of bias

**Bias**	**Authors' judgment**	**Support for judgment**
Is the case definition adequate(Selection)	1	yes, with independent validation
Representativeness of the cases(Selection)	0	potential for selection biases(patients with T2DM)
Selection of Controls(Selection)	0	hospital controls
Definition of Controls(Selection)	1	no hyperuricemia
Comparability of cases and controls on the basis of the design or analysis(Comparability)	2	study controls for age, gender and<br><br>other factors

Ascertainment of exposure(Exposure)	1	secure record (Laboratory examination)
Same method of ascertainment for cases and controls(Exposure)	1	yes
Non-Response rate(Exposure)	0	non respondents described

Hao Wang et al. 2022

Study type	cross-sectional study
Participants	non-diabetic patients<br>Sample size: 7743<br>Mean age in years:45.17 ± 17.10<br>Gender:3806males/3937females<br>Location:US
Outcomes	Main study outcome: It was found that the risk of HU was positively associated with the elevation of TyG, TyG-BMI, TG/HDL-C and METS-IR in a large-scale population of U.S., and TyG-BMI and METS-IR have a better ability to identify HU in both genders.<br>Available outcomes: the associations between the triglyceride glucose index and hyperuricemia.

Risk of bias

Bias	Authors' judgment	Support for judgment
Is the case definition adequate(Selection)	1	yes, with independent validation
Representativeness of the cases(Selection)	1	consecutive or obviously representative series of cases
Selection of Controls(Selection)	1	community controls
Definition of Controls(Selection)	1	no hyperuricemia

Comparability of cases and controls on the basis of the design or analysis(Comparability)	2	study controls for age, gender and<br>other factors
Ascertainment of exposure(Exposure)	1	secure record (Laboratory<br>examination)
Same method of ascertainment for cases and controls(Exposure)	1	yes
Non-Response rate(Exposure)	0	non respondents described

Jiaxin Qi et al. 2023

Study type	retrospective case-control study
Participants	Patients with NAFLD<br>Sample size: 461<br>Mean age in years:NA<br>Gender: 190males/271females<br>Location:China
Outcomes	Main study outcome: TyG index is an independent risk factor for HUA in patients with NAFLD.<br>Available outcomes: the associations between the triglyceride glucose index and hyperuricemia.

Risk of bias

Bias	Authors' judgment	Support for judgment
Is the case definition adequate(Selection)	1	yes, with independent validation
Representativeness of the cases(Selection)	0	potential for selection biases(Patients with NAFLD)

Selection of Controls(Selection)	0	hospital controls
Definition of Controls(Selection)	1	no hyperuricemia
Comparability of cases and controls on the basis of the design or analysis(Comparability)	2	study controls for age, gender and other factors
Ascertainment of exposure(Exposure)	1	secure record (Laboratory examination)
Same method of ascertainment for cases and controls(Exposure)	1	yes
Non-Response rate(Exposure)	0	non respondents described

Qiuhong Li et al. 2022

Study type	cross-sectional study
Participants	patients with diabetic kidney disease<br>Sample size: 6471<br>Mean age in years: 59.11±10.53<br>Gender: 3780males/2691females<br>Location:China
Outcomes	Main study outcome: a significant independent correlation between the TyG index and the risk of hyperuricemia in DKD patients.<br>Available outcomes: the associations between the triglyceride glucose index and hyperuricemia.

Risk of bias

**Bias**	**Authors' judgment**	**Support for judgment**

Is the case definition adequate(Selection)	1	yes, with independent validation
Representativeness of the cases(Selection)	0	potential for selection biases(patients with diabetic kidney disease)
Selection of Controls(Selection)	0	hospital controls
Definition of Controls(Selection)	1	no hyperuricemia
Comparability of cases and controls on the basis of the design or analysis(Comparability)	2	study controls for age, gender and<br><br>other factors
Ascertainment of exposure(Exposure)	1	secure record (Laboratory<br><br>examination)
Same method of ascertainment for cases and controls(Exposure)	1	yes
Non-Response rate(Exposure)	0	non respondents described

Xing Zhen Liu et al. 2019

Study type	cross-sectional study
Participants	adults without self-reported use of antihyperuricemic agents, hypoglycemic agents, or lipid-lowering drugs.<br><br>Sample size:174695<br><br>Mean age in years:45.00±12.20<br><br>Gender: 105162 males/ 69533 females<br><br>Location:China
Outcomes	Main study outcome: TG/HDLc and TyG are strongly associated with hyperuricemia regardless of BMI classification.<br><br>Available outcomes: the associations between the triglyceride<br><br>glucose index and hyperuricemia.

Risk of bias

Bias	Authors' judgment	Support for judgment
Is the case definition adequate(Selection)	1	yes, with independent validation
Representativeness of the cases(Selection)	1	consecutive or obviously representative series of cases
Selection of Controls(Selection)	1	community controls
Definition of Controls(Selection)	1	no hyperuricemia
Comparability of cases and controls on the basis of the design or analysis(Comparability)	1	study controls for age and other factors
Ascertainment of exposure(Exposure)	1	secure record (Laboratory examination)
Same method of ascertainment for cases and controls(Exposure)	1	yes
Non-Response rate(Exposure)	0	non respondents described

Zeinab Ghorbani et al. 2022

Study type	cross-sectional study
Participants	individuals visited the cardiology outpatient clinic<br>Sample size: 1170<br>Mean age in years:NA<br>Gender: 475males/695females<br>Location:Iran

Outcomes	Main study outcome: higher levels of the IR surrogate markers, TyG and TyG-BMI, are associated with higher odds of hyperuricemia in patients with CAD.<br><br>Available outcomes: the associations between the triglyceride glucose index and hyperuricemia.

Risk of bias

Bias	Authors' judgment	Support for judgment
Is the case definition adequate(Selection)	1	yes, with independent validation
Representativeness of the cases(Selection)	0	potential for selection biases(individuals visited the cardiology outpatient clinic)
Selection of Controls(Selection)	0	hospital controls
Definition of Controls(Selection)	1	no hyperuricemia
Comparability of cases and controls on the basis of the design or analysis(Comparability)	2	study controls for age, gender and<br><br>other factors
Ascertainment of exposure(Exposure)	1	secure record (Laboratory<br><br>examination)
Same method of ascertainment for cases and controls(Exposure)	1	yes
Non-Response rate(Exposure)	0	non respondents described

Xuanxia Wu et al. 2023

Study type	cross-sectional study

Participants	a general Population<br>Sample size:32354<br>Mean age in years:NA<br>Gender: 18100 males/ 14254 females<br>Location:China
Outcomes	Main study outcome: The prevalence of HUA was high in the urban health checkup population in Urumqi, Xinjiang, particularly among men and youth.<br>Available outcomes: the associations between the triglyceride glucose index and hyperuricemia.

Risk of bias

**Bias**	**Authors' judgment**	**Support for judgment**
Is the case definition adequate(Selection)	1	yes, with independent validation
Representativeness of the cases(Selection)	1	consecutive or obviously representative series of cases
Selection of Controls(Selection)	1	community controls
Definition of Controls(Selection)	1	no hyperuricemia
Comparability of cases and controls on the basis of the design or analysis(Comparability)	2	study controls for age, gender and other factors
Ascertainment of exposure(Exposure)	1	secure record (Laboratory examination)
Same method of ascertainment for cases and controls(Exposure)	1	yes
Non-Response rate(Exposure)	0	non respondents described

Lei Zhang et al. 2019

Study type	cross-sectional study
Participants	participants of physical examination<br>Sample size: 24438<br>Mean age in years:47.23<br>Gender: 12,557males/11,881females<br>Location:China
Outcomes	Main study outcome: Age-related and gender-stratified differences in the association between high triglyceride and risk of hyperuricemia.<br>Available outcomes: the associations between the triglyceride glucose index and hyperuricemia.

Risk of bias

Bias	Authors' judgment	Support for judgment
Is the case definition adequate(Selection)	1	yes, with independent validation
Representativeness of the cases(Selection)	1	consecutive or obviously representative series of cases
Selection of Controls(Selection)	1	community controls
Definition of Controls(Selection)	1	no hyperuricemia
Comparability of cases and controls on the basis of the design or analysis(Comparability)	2	study controls for age and other factors
Ascertainment of exposure(Exposure)	1	secure record (Laboratory examination)
Same method of ascertainment for cases and controls(Exposure)	1	yes
Non-Response rate(Exposure)	0	non respondents described

Kelibinuer Mutailipu et al. 2024

Study type	cross-sectional study
Participants	Department of Endocrinology at Shanghai Tenth People's Hospital between January 2017 and October 2020.<br>Sample size: 951<br>Mean age in years:31<br>Gender: 406males/545females<br>Location:China
Outcomes	Main study outcome: Sex-based differences in the associations between obesity- and lipid-related indexes and hyperuricemia risk in patients with obesity.<br>Available outcomes: the associations between the triglyceride glucose index and hyperuricemia.

Risk of bias

Bias	Authors' judgment	Support for judgment
Is the case definition adequate(Selection)	1	yes, with independent validation
Representativeness of the cases(Selection)	0	potential for selection biases(patients from department of endocrinology)
Selection of Controls(Selection)	0	hospital controls
Definition of Controls(Selection)	1	no hyperuricemia
Comparability of cases and controls on the basis of the design or analysis(Comparability)	2	study controls for age and other factors
Ascertainment of exposure(Exposure)	1	secure record (Laboratory examination)

Same method of ascertainment for cases and controls(Exposure)	1	yes
Non-Response rate(Exposure)	0	non respondents described

Ruoyu Gou et al. 2024

Study type	cross-sectional study
Participants	data collected from NHANES in the United States and the CHARLS in China.<br><br>Sample size: US:14259 China:4613<br><br>Mean age in years:US:45.92 China:68.52<br><br>Gender:US: 7636males/6636females<br><br>China: 3207males/1406females
Outcomes	Main study outcome: TyG, TyG-BMI, TyG-WHtR and TyG-WC are associated with an increased risk of HUA.<br><br>Available outcomes: the associations between the triglyceride glucose index and hyperuricemia.

Risk of bias

**Bias**	**Authors' judgment**	**Support for judgment**
Is the case definition adequate(Selection)	1	yes, with independent validation
Representativeness of the cases(Selection)	1	consecutive or obviously representative series of cases
Selection of Controls(Selection)	1	community controls
Definition of Controls(Selection)	1	no hyperuricemia
Comparability of cases and controls on the basis of the design or analysis(Comparability)	2	study controls for age and other factors

Ascertainment of exposure(Exposure)	1	secure record (Laboratory examination)
Same method of ascertainment for cases and controls(Exposure)	1	yes
Non-Response rate(Exposure)	0	non respondents described

Linjie Qiu et al. 2024

Study type	cross-sectional study
Participants	data from the NHANES<br>Sample size: 8572<br>Mean age in years:49.2<br>Gender: 4280males/4292females<br>Location:US
Outcomes	Main study outcome: a strong positive connection between TyG and hyperuricemia among adults in the United States.<br>Available outcomes: the associations between the triglyceride glucose index and hyperuricemia.

Risk of bias

**Bias**	**Authors' judgment**	**Support for judgment**
Is the case definition adequate(Selection)	1	yes, with independent validation
Representativeness of the cases(Selection)	1	consecutive or obviously representative series of cases
Selection of Controls(Selection)	1	community controls
Definition of Controls(Selection)	1	no hyperuricemia
Comparability of cases and controls on the basis of the design or analysis(Comparability)	2	study controls for age and other factors

Ascertainment of exposure(Exposure)	1	secure record (Laboratory examination)
Same method of ascertainment for cases and controls(Exposure)	1	yes
Non-Response rate(Exposure)	0	non respondents described

Sethapong Lertsakulbunlue et al. 2024

Study type	cross-sectional study
Participants	Royal Thai Army personnel<br>Sample size: 231286<br>Mean age in years:47.4<br>Gender:206636males/24650females<br>Location:Thailand
Outcomes	Main study outcome: A robust positive association between the TyG index and SUA was illustrated among active-duty RTA personnel.<br>Available outcomes: the associations between the triglyceride glucose index and hyperuricemia.

Risk of bias

**Bias**	**Authors' judgment**	**Support for judgment**
Is the case definition adequate(Selection)	1	yes, with independent validation
Representativeness of the cases(Selection)	0	potential for selection biases(Army personnel)
Selection of Controls(Selection)	0	Army controls
Definition of Controls(Selection)	1	no hyperuricemia
Comparability of cases and controls on the basis of the design or analysis(Comparability)	2	study controls for age and other factors

Ascertainment of exposure(Exposure)	1	secure record (Laboratory examination)
Same method of ascertainment for cases and controls(Exposure)	1	yes
Non-Response rate(Exposure)	0	non respondents described

Li Hongwei et al. 2025

Study type	cross-sectional study
Participants	participants of physical examination<br>Sample size: 14834<br>Mean age in years:50.6<br>Gender: 9788males/5046females<br>Location:China
Outcomes	Main study outcome: The effects and predictive values of novel anthropometric parameters on uric acid levels and hyperuricemia in adults.<br>Available outcomes: the associations between the triglyceride glucose index and hyperuricemia.

Risk of bias

**Bias**	**Authors' judgment**	**Support for judgment**
Is the case definition adequate(Selection)	1	yes, with independent validation
Representativeness of the cases(Selection)	1	consecutive or obviously representative series of cases
Selection of Controls(Selection)	1	community controls
Definition of Controls(Selection)	1	no hyperuricemia
Comparability of cases and controls on the basis of the	2	study controls for age and other factors

design or analysis(Comparability)		
Ascertainment of exposure(Exposure)	1	secure record (Laboratory examination)
Same method of ascertainment for cases and controls(Exposure)	1	yes
Non-Response rate(Exposure)	0	non respondents described

Leixia Wang et al. 2024

Study type	cross-sectional study
Participants	data collected from NHANES<br>Sample size: 7367<br>Mean age in years:51.8<br>Gender: 3561males/3806females<br>Location:US
Outcomes	Main study outcome: A national study exploring the association between triglyceride-glucose index and risk of hyperuricemia events in adults with hypertension.<br>Available outcomes: the associations between the triglyceride glucose index and hyperuricemia.

Risk of bias

Bias	Authors' judgment	Support for judgment
Is the case definition adequate(Selection)	1	yes, with independent validation
Representativeness of the cases(Selection)	1	consecutive or obviously representative series of cases
Selection of Controls(Selection)	1	community controls
Definition of Controls(Selection)	1	no hyperuricemia

Comparability of cases and controls on the basis of the design or analysis(Comparability)	2	study controls for age and other factors
Ascertainment of exposure(Exposure)	1	secure record (Laboratory examination)
Same method of ascertainment for cases and controls(Exposure)	1	yes
Non-Response rate(Exposure)	0	non respondents described

Najmeh Seif et al. 2024

Study type	cross-sectional study
Participants	part of the Mashhad Stroke and Heart Atherosclerotic Disorder (MASHAD) cohort study.<br>Sample size: 6457<br>Mean age in years:48.44<br>Gender: 2579males/3878females<br>Location: Iran
Outcomes	Main study outcome: The association between hyperuricemia and insulin resistance surrogates, dietaryand lifestyle insulin resistance indices in an Iranian population.<br>Available outcomes: the associations between the triglyceride glucose index and hyperuricemia.

Risk of bias

**Bias**	**Authors' judgment**	**Support for judgment**
Is the case definition adequate(Selection)	1	yes, with independent validation
Representativeness of the cases(Selection)	1	consecutive or obviously representative series of cases
Selection of Controls(Selection)	1	community controls

Definition of Controls(Selection)	1	no hyperuricemia
Comparability of cases and controls on the basis of the design or analysis(Comparability)	2	study controls for age and other factors
Ascertainment of exposure(Exposure)	1	secure record (Laboratory examination)
Same method of ascertainment for cases and controls(Exposure)	1	yes
Non-Response rate(Exposure)	0	non respondents described

Yu-Qiang Zuo et al. 2024

Study type	cross-sectional study
Participants	an annual health check-up population<br>Sample size: 6219<br>Mean age in years:39.13<br>Gender:1416males/4803females<br>Location:China
Outcomes	Main study outcome: A national study exploring the association between triglyceride-glucose index and risk of hyperuricemia events in adults with hypertension.<br>Available outcomes: the associations between the triglyceride glucose index and hyperuricemia.

Risk of bias

Bias	Authors' judgment	Support for judgment
Is the case definition adequate(Selection)	1	yes, with independent validation
Representativeness of the cases(Selection)	1	consecutive or obviously

		representative series of cases
Selection of Controls(Selection)	1	community controls
Definition of Controls(Selection)	1	no hyperuricemia
Comparability of cases and controls on the basis of the design or analysis(Comparability)	2	study controls for age and other factors
Ascertainment of exposure(Exposure)	1	secure record (Laboratory examination)
Same method of ascertainment for cases and controls(Exposure)	1	yes
Non-Response rate(Exposure)	0	non respondents described

Table S4 Risk of bias in cohort studies by NOS.

Qiuhong Li et al. 2022

Study type	Cohort study
Participants	patients with diabetic kidney disease<br>Sample size:3634<br>Mean age in years: NA<br>Gender: NA<br>Location: China
Outcomes	Main study outcome: a significant independent association of the TyG index and the risk of hyperuricemia in DKD patients.<br>Available outcomes: the associations between the triglyceride glucose index and hyperuricemia.

Risk of bias

Bias	Authors' judgment	Support for judgment
Representativeness of the exposed cohort (Selection)	0	somewhat representative of the patients without hyperuricemia at the baseline
Selection of the non exposed cohort (Selection)	1	drawn from the same community as the exposed cohort
Ascertainment of exposure (Selection)	1	secure record (eg laboratory examination)
Demonstration that outcome of interest was not present at start of study (Selection)	1	participants did not have hyperuricemia at the baseline
Comparability of cohorts on the basis of the design or analysis (Comparability)	2	study controls for age, gender and other factors
Assessment of outcome (Outcome)	1	secure record (eg laboratory examination)

Was follow up long enough for outcomes to occur (Outcome)	1	Yes (23 months)
Adequacy of follow up of cohorts (Outcome)	1	subjects lost to follow up unlikely to introduce bias, description of those lost

Qing Gu et al. 2020

Study type	Cohort study
Participants	General Population<br><br>Sample size:42387<br><br>Mean age in years: 43.10±12.30<br><br>Gender: 23864males/18523females<br><br>Location: China
Outcomes	Main study outcome: TyG and its integration with obesity indicators have the potential to help risk stratification and prevention of HUA, especially among women.<br><br>Available outcomes: the associations between the triglyceride<br><br>glucose index and hyperuricemia.

Risk of bias

Bias	Authors' judgment	Support for judgment
Representativeness of the exposed cohort (Selection)	1	truly representative of the average<br><br>Chinese adults in the community
Selection of the non exposed cohort (Selection)	1	drawn from the same community as the exposed cohort
Ascertainment of exposure (Selection)	1	secure record (eg laboratory examination)
Demonstration that outcome of interest was not present at start of study (Selection)	1	participants did not have hyperuricemia at the baseline

Comparability of cohorts on the basis of the design or analysis (Comparability)	2	study controls for age, smoking status and other factors
Assessment of outcome (Outcome)	1	secure record (eg laboratory examination)
Was follow up long enough for outcomes to occur (Outcome)	1	Yes (at least 2 years)
Adequacy of follow up of cohorts (Outcome)	1	subjects lost to follow up unlikely to introduce bias, description of those lost

Table S5 Summary of the results using the GRADE (case-control/cross-sectional studies).

The level of triglyceride glucose index in people with hyperuricemia compared with without hyperuricemia.			
Population: Subjects with hyperuricemia vs. normal subjects			
Settings: Twenty-two studies were conducted in Asia, four study was conducted in America.			
Cases: Subjects with hyperuricemia			
Controls: Subjects without hyperuricemia			
Outcomes	OR (95% CI) [a]	No of participants (studies)	Quality of the evidence Comments (GRADE)
hyperuricemia	2.67(2.34,3.04)	591,933 (twenty-five studies)	⊕ ⊕ ⊕ ⊕HIGH [b,c]

GRADE working group grades of evidence

High quality: We are very confident that the true effect lies close to that of the estimate of the effect.

Moderate quality: We are moderately confident in the effect estimate: The true effect is likely to be close to the estimate of the effect, but there is a possibility that it is substantially different.

Low quality: Our confidence in the effect estimate is limited: The true effect may be substantially different from the estimate of the effect.

Very low quality: We have very little confidence in the effect estimate: The true effect is likely to be substantially different from the estimate of effect.

Abbreviations: OR, odds ratio; CI, confidence interval

[a] Results for triglyceride glucose index levels of subjects with hyperuricemia compared with controls; [b] Upgraded by one level due to all the results of the included studies were almost consistent (subjects with hyperuricemia had higher triglyceride glucose index); [c] Upgraded by one level due to a dose-response relationship between hyperuricemia and triglyceride glucose index (The higher triglyceride glucose index, the higher risk of hyperuricemia); GRADE, Grading of Recommendations Assessment, Development and Evaluation system; ⊕, quality of evidence.

Table S6 Summary of the results using the GRADE (cohort studies).

Risk of Hyperuricemia with Different Triglyceride Glucose Index Levels			
Population: Subjects with high level of triglyceride glucose index vs. low level triglyceride glucose index			
Settings: Two studies were conducted in Asia.			
Cases: Subjects with high level of triglyceride glucose index			
Controls: Subjects with low level of triglyceride glucose index			
Outcomes	HR (95% CI) [a]	No of participants (studies)	Quality of the evidence Comments (GRADE)
hyperuricemia	1.68(1.30,2.17)	46,021(two studies)	⊕ ⊕ ⊕ MODERATE [b]

GRADE working group grades of evidence

High quality: We are very confident that the true effect lies close to that of the estimate of the effect.

Moderate quality: We are moderately confident in the effect estimate: The true effect is likely to be close to the estimate of the effect, but there is a possibility that it is substantially different.

Low quality: Our confidence in the effect estimate is limited: The true effect may be substantially different from the estimate of the effect.

Very low quality: We have very little confidence in the effect estimate: The true effect is likely to be substantially different from the estimate of effect.

Abbreviations: HR, hazard ratio; CI, confidence interval

[a] Results for hyperuricemia risk of subjects with higher levels of triglyceride glucose index compared with lower triglyceride glucose index; [b] Upgraded by one level due to all the results of the included studies were almost consistent (subjects with high triglyceride glucose index had high risk of hyperuricemia); GRADE, Grading of Recommendations Assessment, Development and Evaluation system; ⊕, quality of evidence.

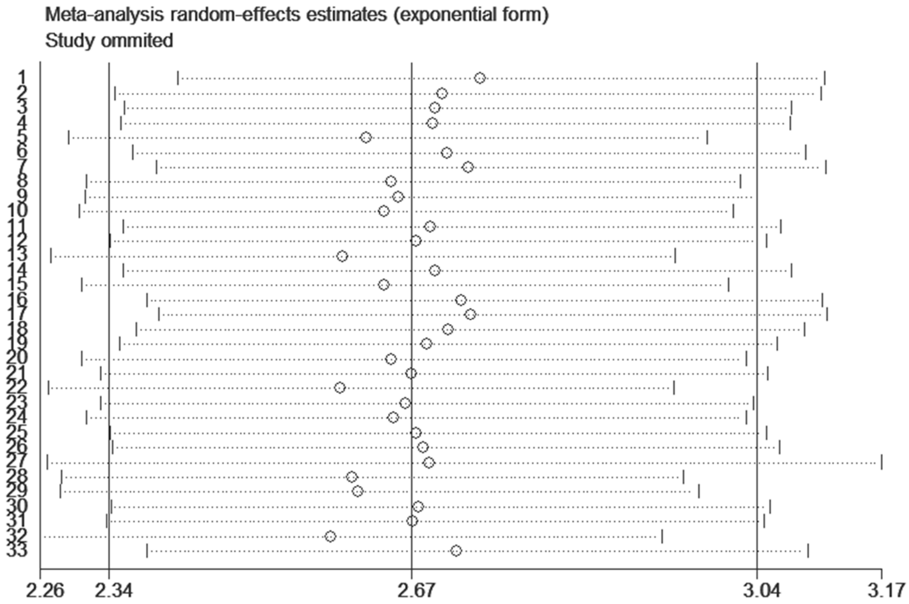

Figure. S1**:** The sensitivity analysis of included studies.
